# Indents

**Published:** 1901-09-15

**Authors:** 


### INDENTS.

**☞ Brief Articles of Technical or Scientific Value will receive notice and comment. Contributions invited.**

COCAIN SOLUTION.—Dr. Duffield recommends a solution of cocain in acetic ether for anesthetizing the pulp by the pressure method. — *International Journal.*

EXTRACTING TEETH WITH THE FINGERS.—An Indianapolis dentist claims to be able to extract all teeth with the fingers only, using no forceps, and with much less pain than the forceps.

A NEW FORM OF TOOTH-BRUSH.—Dr. H. F. Hamilton, of Boston, suggests for persons who excessively use the stiff tooth-brushes, so as to destroy the gum tissue over the prominent teeth, a brush with alternating rows of bristles and badger hair. The badger hairs are longer than the bristles; the bristles serve merely to support the badger hair and keep the brush from matting.

GOLD INLAY MATRIX.—In accessible cavities lay a piece of punk in the cavity first and then place on top of the punk the gold for matrix, and with a suitable burnisher push the gold down into the punk; it will take an approximate shape to the cavity, then remove the punk and the gold can be worked into the cavity without crimping or breaking.—Dr. C. F. Allan, in *International.*

PULVERIZED SOAPSTONE IN THE DENTAL OFFICE.—Have some in a small wide mouth bottle in the operating case to rub on the dam to make it slip between the teeth readily. Dip gutta-percha or cement instruments into it to prevent the material adhering. A pepper box filled with it will be useful in flasking to cause the flasks to separate. It will often make a pair of tight shoes feel more comfortable if dusted into them before putting them on.—Dr. J. G. Harper, in *Dental Review*.

The Treatment of Fermentative Disease of the Infant.—Dr. Kirk says: "Sterilize the oral cavity with a saturative solution of boric acid, to which ten grains of borax are added for each ounce of the solution, or a dilute solution of phenal sodique, applied with a cotton swab. This should be an important part of the infant toilet and repeated as often as may be necessary. For gastro-intestinal irritations, remove the mechanical irritants by laxitives, and correct acidity by suitable chemical substances. Magnesium hydrate is an ideal remedy. It neutralizes the acids of fermentation, forming organic salts of magnesium, which possess mild laxative properties, thus accomplishing in a thorough manner two therapeutic results."—May *Cosmos*.

Preparation for Crowning.—Preparatory treatment of teeth and roots for crown-work includes (in addition to the shaping required to fit them for the reception of the crowns), the bringing about of the healthiest possible condition, not only in the teeth and roots, but the adjacent parts, as the cure of existing lesions, the removal of salivary calculus where necessary, and the adoption of such measures as shall prevent the recurrence of old trouble or the inception of new. There are many teeth and roots which can not be rendered suitable for the successful application of crowns and bridges. Roots which are permeated and softened by decay, exposed or loosened by absorption of the gums and alveoli, or affected with irremediable disease of the investing membranes, should be thus classed. Cases in which abscess with necrosis has extremely impaired the walls of the alveoli are equally intractable.—F. J. Capon, in *Dominion Journal.*

SODIUM PEROXID AND SULPHURIC ACID AS A DISINFECTANT.—Apply the rubber dam to shut out the saliva and wash the tooth with absolute alcohol to remove any micro-organisms which may be attached to it. Make a free and direct opening into the pulp chamber, dip a Donaldson nerve boach into fifty percent sulphuric acid and insert into the pulp cavity, then dip the same broach into the powdered peroxid of sodium and again insert into the pulp chamber. A vigorous re-action at once takes place, the peroxid of sodium is broken up and large volumes of oxygen are released; and being in the uncombined state, combine with the poisonous gases and destroy them. The putrid matter is dissolved and forced out by the effervescence and there is no danger of infected matter being forced through the apex if there is a free opening into the pulp chamber. The tooth is also bleached because all coloring matter is dissolved and the tooth is left clean and sweet. The tooth may be wiped out with cotton, wrapped on a broach and dipped in water and more peroxid of sodium introduced to make sure of counteracting the sulphuric acid.—S. S. McFarland, in *Review.*

